# Experimental and Computational Studies on the Gas-Phase Acidity of 5,5-Dialkyl Barbituric Acids

**DOI:** 10.1021/jasms.1c00123

**Published:** 2021-06-25

**Authors:** Juan Z. Dávalos-Prado, Javier González, Josep M. Oliva-Enrich, Emma J. Urrunaga, Alexsandre F. Lago

**Affiliations:** † Instituto de Química Física “Rocasolano”, CSIC, Serrano 119, E-28006 Madrid, Spain; ‡ Centro de Ciências Naturais e Humanas, Universidade Federal do ABC (UFABC), Avenida dos Estados, 5001, 09210-580, Santo André, São Paulo, Brazil; § Facultad de Ciencias, Universidad Nacional San Antonio de Abad UNSAAC, Avenida de la Cultura No. 733, Wanchac-Cusco 08000, Perú

**Keywords:** gas phase-acidity, alkyl-barbituric acids, extended kinetic Cooks method

## Abstract

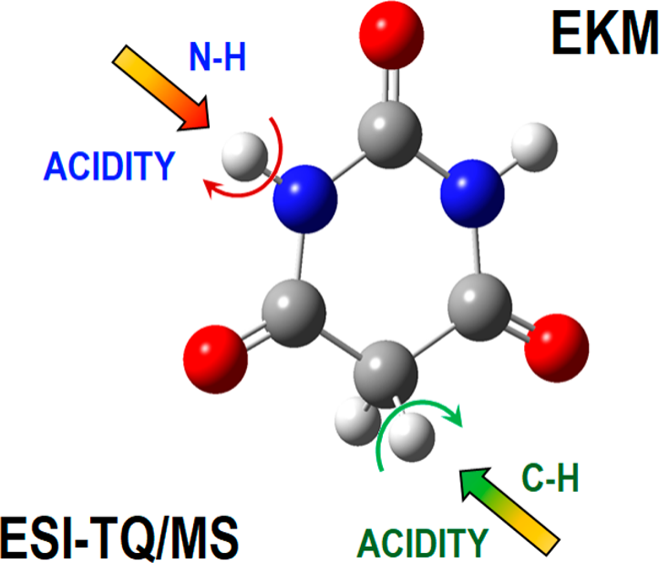

The gas phase acidities (GA) of 5,5-alkylbarbituric acids have been experimentally determined by electrospray ionization-triple quadrupole (ESI-TQ) mass spectrometry and by using the extended kinetic Cooks method (EKCM). The GAs of C–H (1330.9 ± 10.0 kJ mol^–1^) and N–H (1361.5 ± 10.5 kJ mol^–1^) deprotonated sites of bifunctional barbituric acid were determined from the selective production of their corresponding heterodimers. The GA value in the N–H site was confirmed by measuring the GAs of 5,5-dimethyl- and 5,5-diethyl barbituric acids (∼1368 kJ mol^–1^). The experimental results have been rationalized and discussed with the support of quantum chemical calculations with Gaussian-n (G3 and G4) composite methods, which confirmed the excellent consistency of the results.

## Introduction

Barbituric acid (**1**, C_4_H_4_N_2_O_3_) is an organic compound synthesized for the first time in 1864 by Adolf von Baeyer.^1^ Its chemical structure is based on a pyrimidine heterocyclic skeleton and is widely used in the pharmaceutical preparation of several derivatives, *e.g*., the barbiturates, which are well-known for applications in medicinal chemistry due to their biological activities such as hypnotic, sedative, anticonvulsant, anesthetic, antioxidant agent, antibacterial, antifungal, antiviral, and antitumor properties.^2−6^

**Figure 1:**
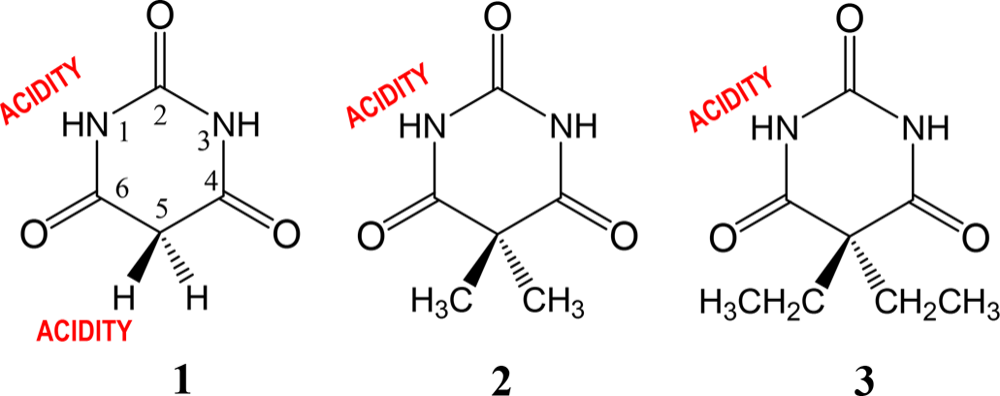
Molecular structure of 5,5-dialkylbarbituric acids, showing the acidic sites.

Barbituric acid, itself, is not biologically active, and the pharmacological properties of its derivatives mainly depend on the side groups attached to the C5 atom of the pyrimidine ring (Figure 1). This C5 position in the molecular structure is considered an active site because it can act either as electrophilic or nucleophilic centers. Some C5-substituted and disubstituted barbituric acids are known to exhibit pharmacological and biological activities.^7^

Another relevant structural feature of barbituric acid is that it contains five additional donor–acceptor sites (three O and two N). Its enolizable hydrogen atoms are bonded to either nitrogen, carbon, or oxygen atoms, resulting in various tautomeric forms. Theoretical and experimental studies on barbituric acid and its derivatives show that the keto-tautomer is the most stable form in the gas phase,^8,9^ as well as in solution,^10,11^ while in the solid state, Schmidt et al.^12^ revealed that the most thermodynamically stable form is a polymorph species formed by the enol tautomer.

The gas phase acidity (GA) of barbituric acid, structure **1** shown in Figure 1, was measured by Kebarle et al.^13−15^ using an equilibrium method (at 500–600 K) with a pulsed electron beam high-pressure ion source mass spectrometer. The corresponding value, GA (1) = 1369.4 ± 8.4 kJ mol^–1^, has been reported in the NIST database^16^ and the authors suggested that it is related to the deprotonation in the carbon–hydrogen (C–H) site.

Electrospray ionization sources (ESI) and mass spectrometry techniques are known as powerful tools to investigate structural, energetic, and other physical chemistry properties of thermolabile biomolecules and other species with low vaporization pressures.^17^ In addition, the Cooks kinetic method (CKM)^18,19^ has been widely employed to study the kinetics and thermodynamic properties of chemical reactions. Fenselau et al.,^20,21^ Wesdemiotis et al.,^22,23^ Armentrout et al.,^24,25^ among others, have improved the KM method by including entropic effects on the competitive dissociations of a mass-selected proton-bound heterodimer (cluster) ions, resulting in the so-called “extended kinetic method, EKM”.

The present work employs electrospray ionization-triple quadrupole (ESI-TQ) mass spectrometry and the extended kinetic method to investigate the deprotonation processes of the bifunctional barbituric acid, **1** (Figure 1), for which the acidic site is located either on nitrogen (N–H) or carbon atoms (C–H). In addition, with the purpose of providing reliable pathways to establish the consistency of the both acidity measurements of **1** and particularly to confirm the N–H acidity, we have selected similar structures such as those of the 5,5-dimethyl barbituric (**2**) and 5,5-diethyl barbituric (**3**, barbital) acids (Figure 1). Each of these structures features only one acidic site (N–H), because the α-carbon site is blocked by, respectively, two-methyl and two-ethyl groups. Furthermore, it is known that the methylation of some N-heterocyclic molecules, such as uracils, produces little effect on the N–H acidity.^26^

By using this approach, which shall be explained in detail throughout the text, we have been able to establish a consistent GA value for **1**, which is discussed and compared with the previously reported value in the literature. In addition, we have determined for the first time the GA values for the structures **2** and **3**.

## Experimental Section

### Chemicals

Barbituric acid (**1**, pyrimidine-2,4,6(1*H*,3*H*,5*H*)-trione), 5,5-diethylbarbituric acid (**2**, dimethylpyrimidine-2,4,6 (1*H*,3*H*,5*H*)-trione), and 5,5-diethylbarbituric acid (**3**, diethylpyrimidine-2,4,6(1*H*,3*H*,5*H*)-trione) samples, as well as the corresponding reference compound acids, were purchased from Merck/Sigma-Aldrich Co. and Alfa Aesar and used without further purification. The suitable reference compounds used in this work were chosen based on their known characteristic thermochemical properties and particularly for presenting similar GA (taken from NIST Database and shown in Table S1 in the Supporting Information) as compared to the target compound studied.

### ESI-TQ Mass Spectrometry Measurements

The experiments were carried out on a triple-quadrupole mass spectrometer (TQ-MS) Agilent/Varian 320 equipped with an electrospray ionization (ESI) source. Approximately 5 × 10^–5^ M 5,5-alkylbarbituric acid and the appropriate reference acid were mixed (in a 1:1 mass ratio), and the mixture was dissolved in methanol–water solution (∼1:1, vol/vol). The solutions were directly infused into the ESI ionization source, operating in negative mode, at a flow rate of 10 μL/min. Air was used as the nebulizer gas. The drying gas temperature and the needle potential of ESI were optimized in the ranges of 100 to 280 °C and −3.5 to −6.0 kV, respectively. The adequate optimization of these parameters was a key factor to determine the acidity in the two different active sites of **1**, as discussed later. The housing temperature and voltage of capillary were maintained at 50 °C and −21 V, respectively. The heterodimeric cluster anions were isolated in the first quadrupole, underwent collision induced dissociation (CID) in the second quadrupole, and finally the resulting fragments were analyzed in the third quadrupole (with the detector potential set at 2 kV). CID experiments were performed using argon as the collision gas (0.4 mTorr) in a center of mass energy (*E*_CM_) range of 0.75 to 4.0 eV. The center of mass energy was calculated as *E*_CM_ = *E*_lab_[*m*/(*M* + *m*)] where *E*_lab_ is the ion kinetic energy in the laboratory frame, *m* is the mass of the collision gas, and *M* is the mass of the heterodimeric cluster ion.

### Kinetic Method (KM) and Extended Kinetic Method (EKM)

The kinetic method (EK), and its improved version (EKM), are based on the rates of competitive dissociation of mass selected cluster ions formed between a sample and a reference compound of known thermochemical properties. Therefore, to determine the gas phase acidity of a molecule AH, for instance, one must generate a proton-bound heterodimer anion [A***H***A_ref(i)_]^−^, where AH is the studied sample and A_ref(i)_H is a set of reference compounds with known GA experimental values.

**Scheme 1:**
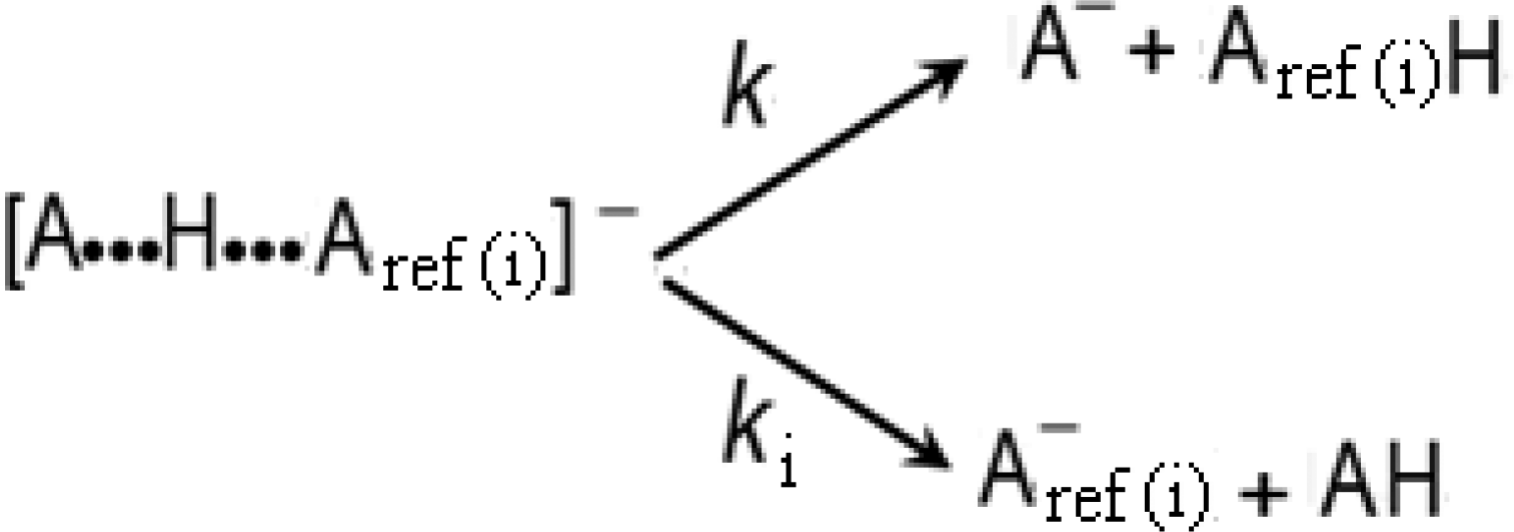
Collision-Induced Dissociations of [A***H***A_ref(i)_]^−^

These clusters are fragmented by collision-induced dissociation (CID) in a collision cell of the spectrometer to yield the corresponding monomeric anions of the sample A^–^ and the reference A_ref(i)_^–^, via the two competitive dissociation channels with rate constants *k* and *k*_*i*_, respectively. If secondary fragmentation is negligible, the abundance ratio of these fragment ions, [A^–^]/[A_ref(i)_^–^], is equal to the ratio of the two dissociation rate constants, *k*/*k*_i_ (Scheme 1) and the logarithm of this ratio is linearly related to the difference in the thermochemical property of interest (see eqs 2 and 3).^27^

The gas-phase acidity (or merely acidity) of a protic acid AH, GA(AH), is defined as the Gibbs free-energy change for reaction 1:
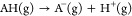
1
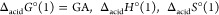
The corresponding enthalpy and entropy changes for reaction 1 are referred to as gas-phase deprotonation enthalpy (Δ_acid_*H*°) and deprotonation entropy (Δ_acid_*S*°), respectively.

With the assumption that there are no reverse activation energy-barriers, the GAs of the sample AH and reference acids A_ref(i)_H are related by the linear eq 2, where *R* is the universal gas constant, and *T*_eff_ is an “effective temperature” related to the excitation energy of the dissociating [A***H***A_ref(i)_]^−^ heterodimers.^28,29^ In the third equality of the eq 2, the free energies (Δ*G*) are expressed in terms of enthalpies (Δ*H*) and entropies (Δ*S*).
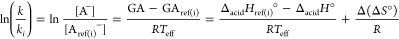
2In order to avoid the correlation of errors between the enthalpy and entropy differences, one must use the statistical procedure developed by Armentrout.^24^ Accordingly, eq 2 is converted into eq 3:
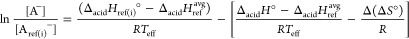
3where Δ_acid_*H*_ref_^avg^ represents the average of the deprotonation enthalpy of the acidic references. The entropic term Δ(Δ*S*°) is the difference of the activation entropies between the two competing dissociation channels which can be expressed as the difference in the deprotonation entropies of the AH and A_ref(i)_H acids, Δ(Δ*S*°) ≈ Δ_acid_*S*° – Δ_acid_*S*_ref(i)_°. If the reference acids have similar deprotonation entropies, the last term can be substituted for the corresponding average entropy, Δ_acid_*S*_ref_^avg^. One can note that eq 3 includes three unknown variables: Δ_acid_*H*°, *RT*_eff_, and Δ_acid_*S*°. These quantities can be obtained from two sets of thermokinetic plots. The first set consists of linear plots of ln([A^–^]/[A_ref_^–^]) vs [Δ_acid_*H*_ref(i)_° – Δ_acid_*H*_ref_^avg^] with 1/*RT*_eff_ as the slope, and the expression between brackets in eq 3 representing the negative *Y*-intercept values. These values (*Y*-intercepts and slopes) are taken and result in the second thermokinetic plot, which yields a straight line with the slope and *Y*-intercept given by [Δ_acid_*H*° – Δ_acid_*H*_ref_^avg^] and −Δ(Δ*S*°), respectively. Thus, deprotonation enthalpy (Δ_acid_*H*°) and deprotonation entropy (Δ_acid_*S*°) can be obtained from these results. Consequently, the gas phase acidity GA value is derived from the general equation Δ*G* = Δ*H* – *T*Δ*S*, where *T* = 298.15 K.

### Computational Details

The quantum chemical calculations were carried out using the Gaussian 09 package.^30^ The geometry optimization and thermochemistry data calculations for the neutral 5,5-alkylbarbituric acids, as well as their corresponding deprotonated ions (see the Supporting Information), were obtained by using the Gaussian-n methods (Gn) at G3 and G4 high levels of theory.^31,32^ These theories have been described in detail in the literature and are well-known to provide very consistent results for a wide range of molecular systems.^33,34^

## Results and Discussion

### Proton-Bound Heterodimer Selection: Case of Barbituric Acid (**1**) with Two Deprotonable Sites (C–N and N–H)

**Figure 2:**
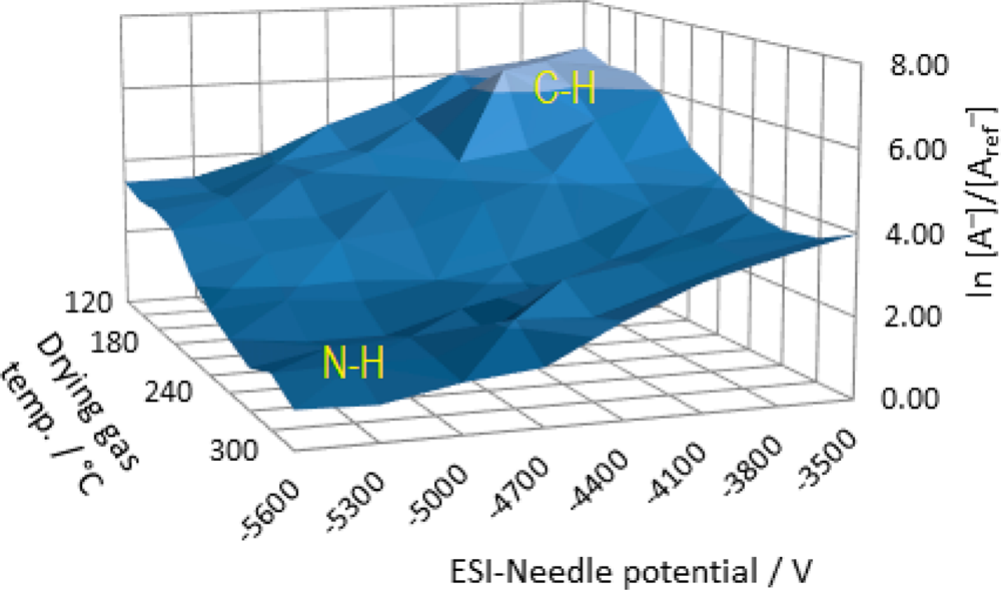
Typical 3D-plot of the natural logarithm of the abundance ratio, ln([A^–^]/[A_ref_^–^]), for AH = **1** and reference compound A_ref_H = 3-chlorobenzoic acid vs drying-gas temperature and needle-potential, both in the ESI source.

In a previous work,^35^ we have applied the extended kinetic method (EKM) to determine the gas phase acidities (GA) of different deprotonable local groups of the same molecule (case of bifunctional hydroxycinnamic acids). The experimental-method development achieved the selective formation of specific hydrogen-bond heterodimers. Varying the drying-gas temperature in the ESI source and the solvents permitted us to generate the binding of reference acids to either local acid-groups of the hydroxycinnamic acids. Thus, the ability to select the gas-phase heterodimer was based particularly on the control of the desolvation process. However, for the barbituric acid case, we have observed that the variation of the drying gas temperature was not enough to select the specific heterodimers. It was also necessary to vary the potential needle of the ESI source. Figure 2 depicts the logarithm of the abundance ratio of ionic fragments, ln([A^–^]/[A_ref(i)_^–^]), in terms of two ESI-parameters: drying-gas temperature and needle-potential. Taking into account the abundance ratio of the ionic fragments, two noteworthy zones can be distinguished in this 3D-plot: (i) “high abundance ratio”, corresponding to the lowest values for both, drying-gas temperature and needle-potential (“soft conditions”), and opposite (ii) “low abundance ratio”, corresponding to the highest values for these considered ESI-parameters (“hardest conditions”).

**Figure 3:**
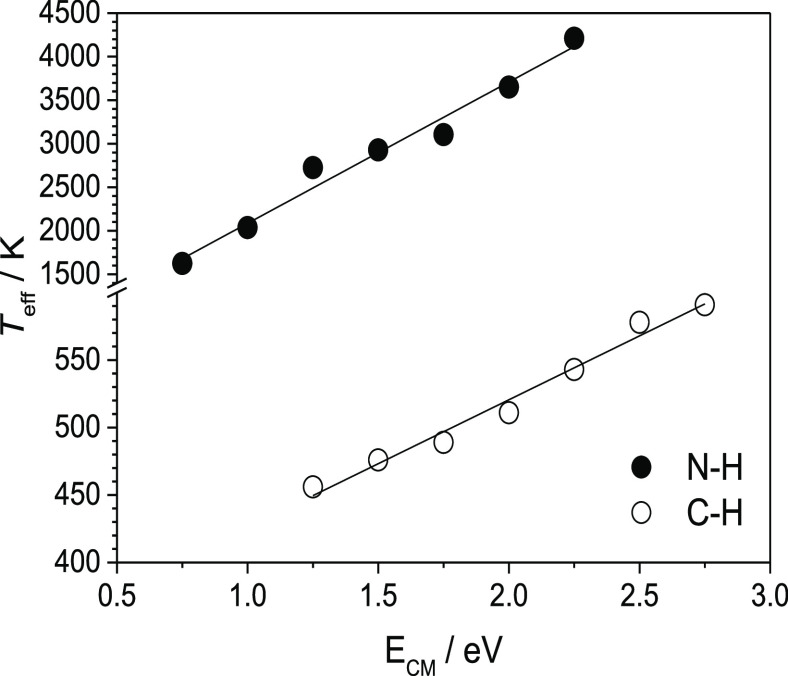
Linear correlations of effective temperature (*T*_eff_) with center mass energies (*E*_CM_) for [A***H***A_ref(i)_]^−^ heterodimers generated by reference acids bonded to either N–H or C–H acid sites of **1**.

The first zone (“soft conditions”) would be related to the production of [A***H***A_ref(i)_]^−^ heterodimers with weak hydrogen-bonds, which involve reference acids bonded to the more acid sites (C–H) of **1**. On the contrary, the second zone (“hardest conditions”) would be related with heterodimers where the reference is bonded to the less acidic sites (N–H) of **1**. In this context, and from a purely experimental point of view, we have found well-defined linear correlations between the “effective temperature” *T*_eff_ (parameter associated with the excitation energy) and the center mass energy *E*_CM_ of the selected dissociating heterodimers (Figure 3), where the low-values of *T*_eff_ would be related to the “soft conditions” while *T*_eff_ high-values would be related to the “hardest conditions”. Accordingly, we can affirm that at low temperatures only the C–H heterodimers are desolvated, and at high temperatures these heterodimers are rather selectively removed from the mixture by dissociation.

As mentioned above, we have established consistent experimental conditions to select heterodimers of the bifunctional barbituric acid attached to its C–H (“soft conditions”) or N–H (“hardest conditions”) groups with the purpose to determine the corresponding GA values. For the first case, we have chosen four compounds with low-GA reference acids (1328.0–1343.5 kJ mol^–1^): trifluoroacetic acid, salicylic acid, 4-nitrophenol, and 4-nitrobenzoic acid. Whereas for the second case, were chosen also four compounds but with high- GA reference acids (1360.6–1390.8 kJ mol^–1^): 3-trifluoromethyl benzoic acid, 3-chloro benzoic acid, 2-*t*-butyl benzoic acid, and 2-methyl benzoic acid. The corresponding thermochemical quantities for all these compounds are listed in Table S1 of the Supporting Information. The CID abundance ratios of the product ions were recorded, for both cases, at seven collision energies (*E*_CM_): from 1.25 to 2.75 eV for C–H and 0.75 to 2.25 eV for N–H sites.

**Figure 4:**
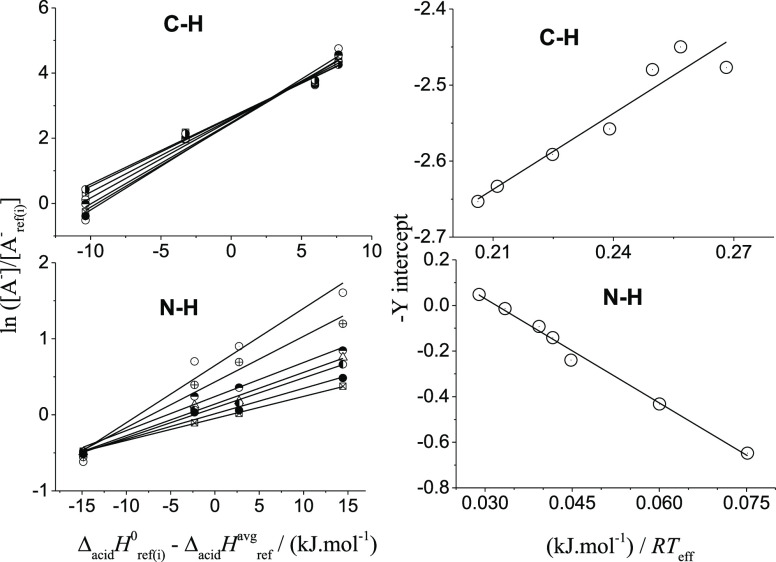
First (left) and second (right) sets of thermokinetic plots for the CH (up) and NH (down) groups of bifunctional barbituric acid (**1**), using CID data of selected [A***H***A_ref(i)_]^−^ heterodimers. The symbols of left graphs correspond to seven collision energies (*E*_CM_).

For each case, the logarithms of the abundance ratios, ln([A^–^]/[A_ref_^–^]), are plotted against the values of [Δ_acid_*H*_ref(i)_° – Δ_acid_*H*_ref_^avg^] (1st thermokinetic plots, depicted in Figure 4,left). The linear regression analysis of these data yield straight lines with slopes 1/*RT*_eff_ and the *Y*-intercepts of −[(Δ_acid_*H*^0^ – Δ_acid_*H*_ref_^avg^)/*RT*_eff_ – Δ(Δ*S*^0^)/*R* ].

These data are finally plotted (2nd thermokinetic plots, on the right of Figure 4). The deprotonation enthalpy Δ_acid_*H*° and entropy (Δ_acid_*S*°) for both the C–H and N–H sites of **1** were derived from linear-fit data of the second thermokinetic plots. As suggested by Armentrout,^24^ the uncertainties of the first thermo-kinetic plots were included in the second one. Our experimentally measured Δ_acid_*H*°, Δ_acid_*S*°, and Δ_acid_*G*° (= GA) were thus obtained from the Δ_acid_*H*_ref_^avg^, Δ_acid_*S*_ref_^avg^, and their uncertainties. The corresponding results are shown in Table 1.

**Table 1:** Experimental Deprotonation Enthalpies (Δ_acid_*H*°), Entropies (Δ_acid_*S*°), and Gas Phase Acidities (GA) of 5,5-Alkylbarbituric Acids

				GA_theor_^a^^,^^d^	
	Δ_acid_*H*^0^^a^	Δ_acid_*S*^0^^b^	GA^a^^,^^c^	G3	G4
barbituric acid (C–H)	1368.6 ± 10.0	125.5 ± 8.4	1330.9 ± 10.0	1332.6	1334.7
barbituric acid (N–H)	1389.1 ± 10.5	93.7 ± 8.4	1361.5 ± 10.5	1364.4	1369.8
5,5-dimethylbarbituric acid, **2**	1404.2 ± 8.8	106.7 ± 8.4	1369.4 ± 8.8	1371.5	1369.0
5,5-diethylbarbituric acid, **3**	1397.9 ± 8.8	98.7 ± 8.4	1368.2 ± 8.8	1367.7	1366.5

a All values are given in kJ mol^–1^.

b In J mol^–1^ K^–1^.

c Derived from equation Δ*G* = Δ*H* – *T*Δ*S*, with *T* = 298.15 K.

d GA theoretical values calculated at G3 and G4 levels of theory.

The resulting slopes, *Y*-intercepts, effective temperatures (*T*_eff_), as well as the resulting uncertainties are summarized in Table S3 (Supporting Information). The comparison between the experimental and calculated thermochemical properties demonstrates good agreement (particularly with G3 methodology), being the deviation less than their corresponding experimental uncertainties (see Table 1).

It should be pointed out that the ancillary GA = 1369.4 ± 8.4 kJ mol^–1^ value reported by Cumming and Kebarle^15^ and currently recommended in the NIST database^16^ is much closer to the GA value of **1** deprotonated in N–H than in C–H site, as originally claimed. Therefore, our present results suggest that this value and assignment should be revised.

### Acidity of 5,5-Dimethylbarbituric (**2**) and 5,5-Diethylbarbituric (**3**) Acids

**Figure 5:**
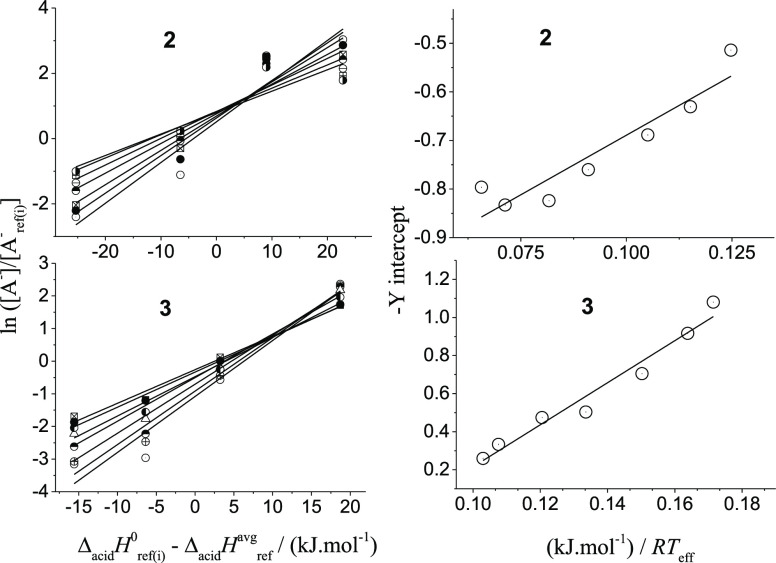
First (left) and second (right) sets of thermokinetic plots for monofunctional 5,5-dimethyl barbituric (**2**, upper) and 5,5-diethylbarbituric acids (**3**, lower), using CID data of [A***H***A_ref(i)_]^−^ heterodimers. The symbols of the left graphs correspond to seven collision energies (*E*_CM_).

Four compounds with GAs ranging from 1343.5 to 1390.3 kJ mol^–1^ (listed in Table S1) were chosen as references for measuring acidity properties of both **2** (4-nitrophenol, 3-trifluorobenzoic acid, 4-hydroxybenzoic acid, 2-methyl benzoic acid) and **3** (4-nitrophenol, 4-nitro-5-methylphenol, 3-trifluoromethyl benzoic acid, 4-hydroxy benzoic acid). The CID abundance ratios of the product ions were recorded, for both compounds, at seven collision energies (*E*_CM_): from 1.0 to 4.0 eV. The EKM results were analyzed in the same manner as for **1** and are shown in Figure 5. The final derived thermochemical results are listed in Table 1. It is important to note that both, **2** and **3**, are deprotonated in their N–H groups, and their corresponding thermodynamical acidity values (GA, Δ_acid_*H*° and Δ_acid_*S*°) are very close, indicating that the methylation of barbituric acid yields little effect on the N–H acidity. These results are also extended for **1**, deprotonated at the same site. Finally, it is worth mentioning that a particularly good agreement has been achieved between the experimental and calculated acidity values (deviation less than 9 kJ mol^–1^), using the G3 and G4 composite methods.

## Conclusions

ESI-TQ mass spectrometry and the extended kinetic Cooks method (EKCM) have been employed to determine the gas phase GA, Δ_acid_*H*° and Δ_acid_*S*° for 5,5-alkylbarbituric acids: barbituric (**1**), 5,5-dimethyl barbituric (**2**), and 5,5-diethyl barbituric (**3**) acids. In the case of bifunctional barbituric acid, it was necessary to generate selective formation of heterodimers in the ESI source, varying both the drying gas temperature and the potential needle. These particular conditions made it possible to obtain binding of reference acids to either the C–H (“soft conditions”) or N–H (“hardest conditions”) acidic groups of **1**. Thus, we have found that **1** deprotonated at C–H (1330.9 ± 10.0 kJ·mol^–1^) is 30.6 kJ mol^–1^ more acid than that deprotonated at the N–H site (1361.5 ± 10.5 kJ mol^–1^), and this last value is close to the GA values of **2** (1369.4 ± 8.8 kJ mol^–1^) and **3** (1368.2 ± 8.8 kJ mol^–1^), both also deprotonated at N–H groups. Overall, a very good agreement between experimental and computational (obtained at G3 and G4 levels of theory) results has been achieved in this work.

## Supporting Information Available

Gas-phase thermochemical data (experimental and G-n theoretical calculations) and CID-experimental and EKM-treatment data (PDF)
