# Supplementary material for: Experimental and Computational Studies on the Gas-Phase Acidity of 5,5-Dialkyl Barbituric Acids
Source: J Am Soc Mass Spectrom. 2021 Jun 25;32(8):2162–7. doi: 10.1021/jasms.1c00123 (PMC8495898; doi:10.1021/jasms.1c00123)
Supplement: Supplementary file 1 — js1c00123_si_001.pdf [file js1c00123_si_001.pdf]

Experimental and computational studies on the gas-phase acidity of 5,5-dialkyl barbituric acids.

Juan Z. Dávalos-Prado[1]*, Javier González[1], Josep M. Oliva-Enrich[1], Emma J. Urrunaga[2], Alexsandre F. Lago[3]*

[1]Instituto de Química Física "Rocasolano", CSIC, Serrano 119, E-28006 Madrid, Spain

[2] Facultad de Ciencias, Universidad Nacional de San Antonio Abad de Cusco,UNSAAC. Av. de la Cultura N° 733, Wanchac-Cusco, Perú.

[3] Centro de Ciências Naturais e Humanas, Universidade Federal do ABC (UFABC), Av. dos Estados, 5001, 09210-580, Santo André, SP, Brazil.

Supporting Information

Table of Contents

Table S1. Gas-phase thermochemical quantities of reference acids used to determine the acidity of 5,5-alkyl barbituric acids: barbituric- (**1**), 5,5-dimethylbarbituric- (**2**) and 5,5-diethylbarbituric (**3**) acids. S3

Table S2. Values of ln([A^{-}]/[$A_{ref(i)}^{-}$]) of CID products of heterodimer [$A\text{-}H\text{-}A_{ref(i)}]^{-}$ of AH= 5,5-alkyl barbituric acids and their corresponding Reference acids, obtained at different collision center of mass -energies E_{CM}. S4

Table S3. Values of the linear-fits obtained from the 1st- themokinetic plots at seven E_{CM} collision energies. S5

Table S4. Enthalpies at 298 K, H_{298}, and Gibbs Energies at 298 K, G_{298} calculated at G3 and G4 level of theory. All Values in Hartrees. S6

Table S1. Gas-phase thermochemical quantities of reference acids used to determine the acidity of 5,5-alkyl barbituric acids:[a] barbituric- (**1**), 5,5-dimethylbarbituric- (**2**) and 5,5-diethylbarbituric (**3**) acids.

Compound of Reference	$\Delta_{acid}G^{0}_{ref(i)}$ [b]	$\Delta_{acid}H^{0}_{ref(i)}$ [b]	$\Delta_{acid}S^{0}_{ref(i)}$ [c,d]	Alkyl Barbituric acids			
				**1 CH**	**1 NH**	**2**	**3**
Trifluoroacetic acid	317.4 ± 2.0	323.8 ± 2.9	21.5 ± 2.0	X			
Salicylic acid	317.8 ± 2.0	325.5 ± 2.2	25.8 ± 2.0	X			
4-Nitrophenol	320.9 ± 2.0	327.7 ± 2.1	22.8 ± 2.0	X		X	X
4-Nitro benzoic acid	321.1 ± 2.0	328.1 ± 2.2	23.5 ± 2.0	X			
4-Nitro-5-methylphenol	322.6 ± 2.0	329.9 ± 2.1	24.5 ± 2.0				X
3-Trifluoromethyl benzoic acid	325.2 ± 2.0	332.2 ± 2.1	23.5 ± 2.0		X	X	X
3-Chloro benzoic acid	328.2 ± 2.0	335.2 ± 2.1	23.5 ± 2.0		X		
4-Hydroxy benzoic acid	328.9 ± 2.0	335.9 ± 2.1	23.5 ± 2.0			X	X
2-*t*-Butyl benzoic acid	329.3 ± 2.0	336.4 ± 2.2	23.8 ± 2.0		X		
2-Methyl benzoic acid	332.4 ± 2.0	339.2 ± 2.2	22.8 ± 2.0		X	X	
Average values			$\Delta_{acid}H^{avg}_{ref}$ [e]	326.5 ± 2.2	335.8 ± 2.5	334.0 ± 2.2	331.7 ± 2.2
			$\Delta_{acid}S^{avg}_{ref}$ [d]	23.0 ± 2.0	23.8 ± 2.0	23.6 ± 2.0	23.7 ± 2.0

[a] Taking from NIST Chemistry Webbook, NIST Standard Reference Database, http://webbook.nist.gov. [b] In kcal mol^{-1}. [c] In cal mol^{-1} K^{-1}. [d] It is assumed to have ± 2.0 cal·mol^{-1}·K^{-1} uncertainty. [e] Since uncorrelated uncertainty with weighting factor (equals to $1/s_i^2$ where s_i = uncertainty experimental of i-datum) is almost ± 1 kcal·mol^{-1} while standard deviation, depending on the case, it can be up to ± 4.9 kcal·mol^{-1}, we decided to considerer as accuracy of the consigned value, to the average of uncertainty experimental values.

Table S2. Values of ln($[A^-]/[A_{ref(i)}^-]$) of CID products of heterodimer $[A\text{-}H\text{-}A_{ref(i)}]^-$ of AH= 5,5-alkyl barbituric acids and their corresponding Reference acids, obtained at different collision center of mass -energies E_{CM}.

**1** C-H	**ln($[A^-]/[A_{ref(i)}^-]$)**						
E_{CM} /eV / $A_{ref(i)}H$	**1.25**	**1.50**	**1.75**	**2.00**	**2.25**	**2.50**	**2.75**
Trifluoroacetic acid	-0.518	-0.389	-0.263	-0.017	0.117	0.325	0.431
Salicylic acid	2.037	1.964	1.975	1.987	2.130	2.178	2.143
4-Nitrophenol	3.632	3.661	3.675	3.721	3.768	3.719	3.782
4-Nitro benzoic acid	4.756	4.563	4.532	4.540	4.351	4.310	4.256
**1** C-N							
	**0.75**	**1**	**1.25**	**1.5**	**1.75**	**2**	**2.25**
3-Trifluoromethyl benzoic acid	-0.619	-0.555	-0.483	-0.493	-0.507	-0.518	-0.478
3-Chloro benzoic acid	0.703	0.393	0.241	0.126	0.063	0.031	-0.106
2-*t*-Butyl benzoic acid	0.902	0.694	0.358	0.179	0.151	0.059	0.018
2-Methyl benzoic acid	1.606	1.198	0.845	0.754	0.662	0.484	0.375
**2**							
	**1**	**1.5**	**2**	**2.5**	**3**	**3.5**	**4**
4-Nitrophenol	-2.403	-2.208	-2.036	-1.603	-1.358	-1.126	-1.007
3-Trifluoromethyl benzoic acid	-1.114	-0.635	-0.300	-0.067	0.067	0.237	0.226
4-Hydroxy benzoic acid	2.538	2.503	2.512	2.290	2.438	2.295	2.182
2-Methyl benzoic acid	3.037	2.864	2.580	2.421	2.149	1.926	1.784
**3**							
	**1**	**1.5**	**2**	**2.5**	**3**	**3.5**	**4**
4-Nitrophenol	-3.157	-3.074	-2.621	-2.223	-2.044	-1.869	-1.691
4-Nitro-5-methylphenol	-2.962	-2.469	-2.227	-1.767	-1.562	-1.207	-1.184
3-Trifluoromethyl benzoic acid	-0.569	-0.439	-0.247	-0.204	-0.251	0.008	0.122

4-Hydroxy benzoic acid	2.365	2.317	2.276	2.182	1.960	1.736	1.714

Table S3. Values of the linear-fits obtained from the 1st- themokinetic plots at seven E_{CM} collision energies

	E_{CM}/eV[(a)]	1.25	1.50	1.75	2.00	2.25	2.50	2.75
Barbituric acid 1(C-H)	T_{eff} /K	449 ± 64	469± 54	482 ± 52	503± 47	535 ± 47	570 ± 49	583 ± 36
	Y-intercept	2.48 ±0.27	2.45 ± 0.21	2.48 ± 0.19	2.56 ±0.16	2.59 ±0.14	2.63 ± 0.13	2.65 ±0.09
	Slope	0.97 ± 0.17	0.98 ± 0.13	0.98 ±0.13	0.99±0.08	0.99±0.08	0.99± 0.08	1.00±0.04
		**0.75**	**1.00**	**1.25**	**1.50**	**1.75**	**2.00**	**2.25**
Barbituric acid 1(N-H)	T_{eff} /K	1600± 232	2004± 240	2685± 271	2887± 262	3060± 210	3598± 415	4151± 68
	Y-intercept	0.65 ±0.11	0.43 ± 0.07	0.24 ± 0.05	0.14 ±0.04	0.09 ±0.03	0.01 ±0.04	-0.05 ±0.00
	Slope	0.31± 0.01	0.25± 0.01	0.19±0.00	0.17±0.00	0.16±0.00	0.14± 0.00	0.12±0.00
		**1.0**	**1.5**	**2.0**	**2.5**	**3.0**	**3.5**	**4.0**
5,5-Dimethylbarbituric acid (2)	T_{eff} /K	964±196	1045±193	1145±234	1322±253	1473±387	1687±479	1831±545
	Y-intercept	0.51±0.46	0.63±0.38	0.69±0.38	0.76±0.31	0.82±0.38	0.83±0.36	0.80±0.35
	Slope	0.52±0.03	0.48±0.02	0.44±0.02	0.38±0.02	0.34±0.02	0.30±0.02	0.27±0.02
5,5-Diethylbarbituric Acid (3)	T_{eff} /K	702±109	734±77	801±101	901±104	998 ±108	1118±61	1168±94
	Y-intercept	-1.08±0.34	-0.92±0.22	-0.70± 0.24	-0.50± 0.20	-0.47±0.17	-0.33±0.08	-0.26±0.11
	Slope	0.72±0.03	0.69±0.02	0.63±0.02	0.56±0.02	0.50±0.01	0.45±0.01	0.43±0.01

Table S4. Enthalpies at 298 K, H_{298}, and Gibbs Energies at 298 K, G_{298} calculated at G3 and G4 level of theory. All Values in Hartrees.

	Neutral		Anion	
	**G3**			
	H_{298}	G_{298}	H_{298}	G_{298}
Barbituric acid (**1**)	-489.776279	-489.817302	(C) -489.259814<br>(N) -489.246093	-489.299646<br>-489.287656
5,5-Dimethylbarbituric acid (**2**)	-568.324748	-568.373030	-567.793115	-567.840581
5,5-Diethylbarbituric acid (**3**)	-646.869467	-646.923361	-646.338483	-646.392336
	**G4**			
Barbituric acid (**1**)	-489.865335	-489.907002	(C) -489.348767<br>(N) -489.336374	-489.388642<br>-489.375240
5,5-Dimethylbarbituric acid (**2**)	-568.428038	-568.476067	-567.896413	-567.944697
5,5-Diethylbarbituric acid (**3**)	-646.986509	-647.040715	-646.455581	-646.510160
